# Supplementary material for: Surgical Approach to Liver Metastases in GEP-NET in a Tertiary Reference Center
Source: Cancers (Basel). 2023 Mar 29;15(7):2048. doi: 10.3390/cancers15072048 (PMC10093386; doi:10.3390/cancers15072048)
Supplement: Supplementary file 1 [file cancers-15-02048-s001.zip › cancers-2219763-supplementary.pdf]

cancers

MDPI

Supplementary Table S1. Characteristics of patients who underwent liver-directed surgery for NELM according to surgical approach.

		MILS (*n* = 13)	OLS (*n* = 39)	*p*
Gender[1]	Female<br>Male	6 (46%)<br>7 (54%)	24 (62%)<br>15 (38%)	0.331
Age (years)[2]		57 (46 – 73)	60 (21 – 80)	0.664
BMI (kg/m^2)[2]		25 (20 – 36)	25 (19 – 34)	0.715
ASA[1]	2<br>3	5 (38%)<br>8 (62%)	27 (69%)<br>12 (31%)	**0.048**
Localization of primary[1]	Pancreas<br>Small intestine	6 (46%)<br>7 (54%)	17 (44%)<br>22 (56%)	0.872
Appearance of metastases[1]	Synchronous<br>Metachronous	10 (77%)<br>3 (23%)	28 (72%)<br>11 (28%)	0.718
Indication[1]	Curative<br>Debulking	5 (38%)<br>8 (62%)	17 (44%)<br>22 (56%)	0.746
Duration of surgery (minutes)[2]		285 (130 – 504)	265 (90 – 575)	0.767
Extent of surgery[1]	Major resection<br>Minor resection	3 (23%)<br>10 (77%)	14 (36%)<br>25 (64%)	0.636
Simultaneous resection of primary[1]		4 (31%)	19 (49%)	0.259
ICU[1]		11 (85%)	33 (85%)	1.0
Length of ICU stay[2]		1 (0 – 6)	1 (0 – 35)	0.702
Length of hospital stay[2]		10 (7 – 20)	12 (6 – 132)	0.100
90-day complications[1]		6 (46%)	18 (46%)	1.0

90-day major complications[1]		5 (38%)	15 (38%)	1.0
90-day mortality[1]		0 (0%)	0 (0.0%)	1.0
Resection of primary[1]		12 (92%)	39 (100%)	0.080
Grading primary[1]	G1<br>G2<br>G3	4 (31%)<br>8 (61%)<br>1 (8%)	14 (36%)<br>25 (64%)<br>0 (0%)	0.214
Grading hepatic metastases[1]	G1<br>G2<br>G3	4 (31%)<br>8 (61%)<br>1 (8%)	15 (38%)<br>20 (52%)<br>4 (10%)	0.813
R status[1]	R0<br>R1<br>R2	4 (31%)<br>1 (8%)<br>8 (61%)	17 (44%)<br>0 (0%)<br>22 (56%)	0.179
Neoadjuvant therapy[1]		3 (23%)	19 (49%)	0.143
Adjuvant therapy[1]		11 (85%)	30 (77%)	0.678
>1 adjuvant therapy modalities[1]		6 (46%)	20 (51%)	0.749
>2 adjuvant therapy modalities[1]		3 (23%)	12 (31%)	0.596

[1] Count (percentage); [2] Median (range); NELM, neuroendocrine liver metastases; MILS, Minimally invasive liver surgery; OLS, open liver surgery; BMI, Body Mass Index; ASA, American Society of Anesthesiologists; ICU, Intensive Care Unit

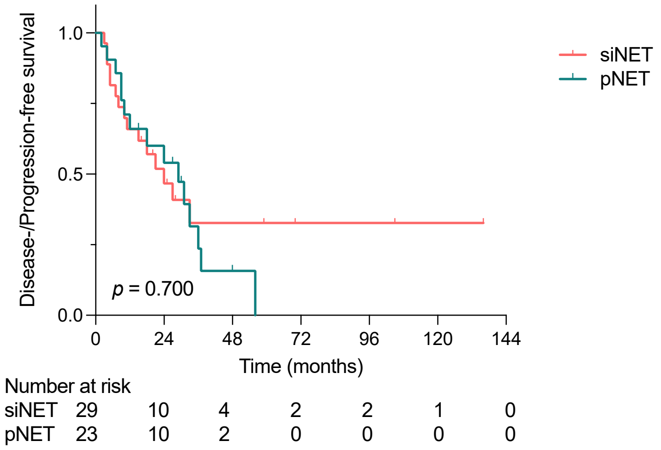

(a)

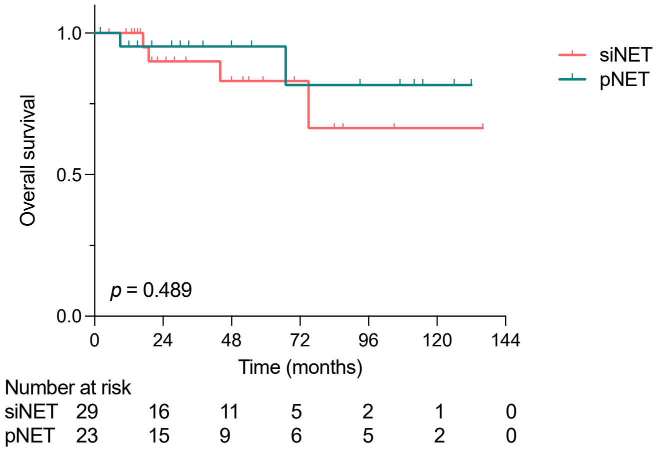

(b)

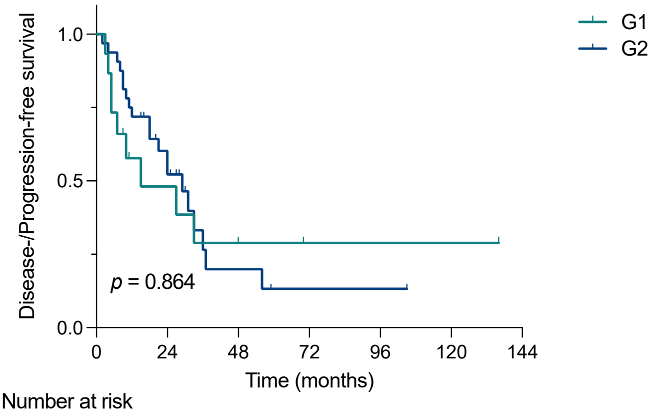

Number at risk

G1	18	5	3	1	1	1	0
G2	33	15	3	1	1	0	0

(c)

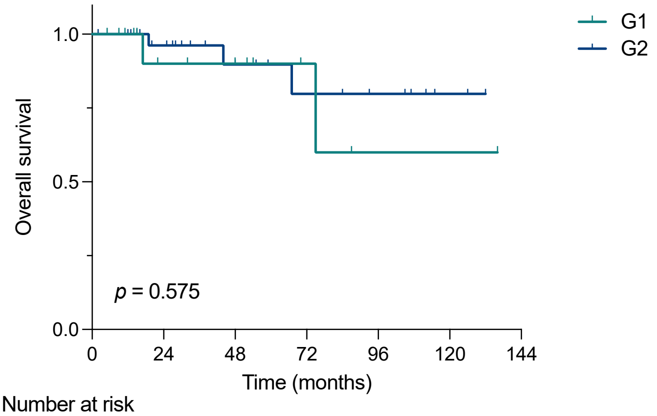

Number at risk

G1	18	8	7	3	1	1	0
G2	33	23	13	8	6	2	0

(d)

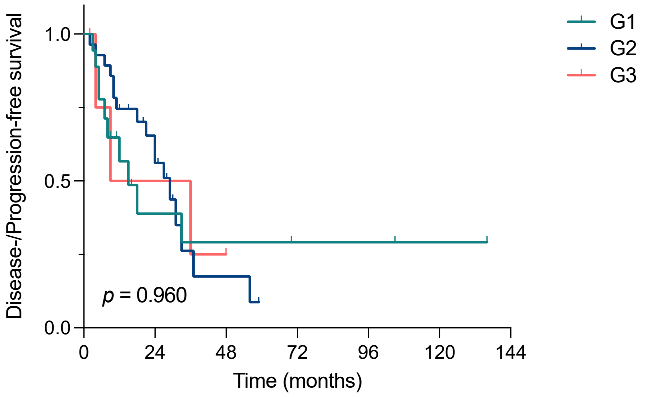

Number at risk

G1	19	4	3	2	2	1	0
G2	28	14	2	0	0	0	0
G3	5	2	1	0	0	0	0

(e)

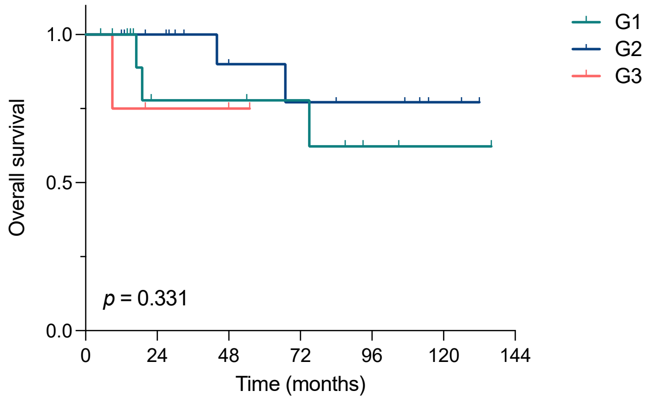

Number at risk

G1	19	8	7	5	2	1	0
G2	28	21	11	6	5	2	0
G3	5	2	2	0	0	0	0

(f)

Supplementary Figure S1. Disease / Progression free and overall survival according to primary tumor site, grading of primary tumor and hepatic metastases. Different primary tumor sites did not show significant differences in (a) DFS / PFS nor (b) OS; (c) DFS / PFS and (d) OS did not differ significantly between primary tumor gradings focusing on G1 and G2 tumors; Different gradings of hepatic metastases had no significant effect on (e) DFS /PFS nor (f) OS. Survival analyses were performed using Kaplan-Meier method.
